# Evaluation of liver function using Gd-EOB-DTPA-enhanced MRI with T1 mapping

**DOI:** 10.1186/s12880-023-01028-z

**Published:** 2023-06-05

**Authors:** Boyang Ma, Hui Xu, Xinru Wu, Wenyan Zhu, Xinjun Han, Jiahui Jiang, Yuxin Wang, Dawei Yang, Hao Ren, Zhenghan Yang

**Affiliations:** Department of Radiology, Beijing Friendship Hospital, Capital Medical University, No. 95 Yongan Road, West District, Beijing, 100050 China

**Keywords:** Liver function, Magnetic resonance imaging, Gd-EOB-DTPA, T1 mapping

## Abstract

**Purpose:**

To evaluate the value of MRI T1 mapping with Gd-EOB-DTPA for assessing liver function.

**Methods:**

Seventy-two patients who underwent Gd-EOB-DTPA-enhanced MRI for focal liver lesions at Beijing Friendship Hospital from August 2020 to March 2022 were prospectively enrolled, and variable-flip-angle T1 mapping was performed before and 20 min after enhancement. The Child–Pugh (C-P) score and albumin-bilirubin (ALBI) grade of liver function were assessed using the clinical data of the patients. Correlation analysis was used to evaluate the correlation between T1 mapping parameters and liver function grading and laboratory tests. Nonparametric tests were used to compare the differences among different liver function groups. The liver function classification efficiency of each image index was evaluated with receiver operating characteristic (ROC) curves.

**Results:**

T1post was positively correlated with the C-P grade and the ALBI grade (*r* = 0.717 and *r* = 0.652). ΔT1 was negatively correlated with the C-P grade and the ALBI grade (*r* = -0.790 and *r* = -0.658). T1post and ΔT1 significantly differed among different liver function grades (*p* < 0.05). For the C-P grade, T1post and ΔT1 were significantly different between each pair of groups (*p* < 0.05), and ΔT1 had a better diagnostic efficiency than T1post. For the ALBI grade, ΔT1 and T1post were significantly different between the NLF and ALBI1 groups (*p* < 0.05), and ΔT1 had a better diagnostic efficacy than T1post. T1post significantly differed between the ALBI1 and ALBI2 + 3 groups (*p* < 0.05), while ΔT1 had a weak ability to differentiate between these two groups.

**Conclusion:**

T1post and ΔT1 were strongly correlated with the two liver function grades and can be noninvasive imaging indexes for evaluating liver function.

## Introduction

In clinical work, the preoperative liver function reserve of patients with liver disease determines whether and to what extent patients can tolerate surgery [1]. In patients with liver cancer, assessments of residual liver function are critical to minimizing the risk of postoperative liver failure [2]. However, in the clinical treatment of liver-occupying masses, the monitoring of liver function changes and preoperative evaluations of liver function reserve can improve the curative effect of medical treatment for tumors as well as postoperative recovery and prognosis [3]. Therefore, accurate evaluations of liver function play a crucial role in the treatment of liver disease and prognosis prediction. In daily practice, the severity of liver disease and liver function often depend on the clinical symptoms and blood biochemical parameters of the disease. Currently, the Child–Pugh (C-P) score and albumin-bilirubin (ALBI) [4, 5] grade are commonly used in the clinic to evaluate liver function, and the latter has a good correlation with the indocyanine green retention rate at 15 min (ICG-R15) [6]. However, these methods include two subjective factors, ascites and hepatic encephalopathy, and some indicators are susceptible to the effects of treatment and other factors, so it is difficult to accurately reflect changes in liver function before and after surgery. Conventional imaging (ultrasound, computed tomography (CT), magnetic resonance imaging (MRI)) cannot quantitatively assess liver function. Previous studies have shown that bile duct excretion, signal intensity (SI), hepatocyte uptake rate, and biliary-to-paravertebral muscle signal intensity ratio (SIR) can be used to estimate liver function [7–9]. The T1 mapping sequence based on gadolinium disodium-enhanced MRI is not affected by the device itself and can reflect the true T1 value of tissues [10], thus providing certain value in the quantitative evaluation of liver function [11]. Previous T1 mapping studies have mostly combined this approach with other methods, but there have been few studies evaluating the various quantitative parameters and ALBI grade for assessing liver function. This study explored the value of Gd-EOB-DTPA-enhanced T1 mapping on MRI for evaluating liver function, with different clinical liver function grades serving as the reference.

## Statement

This study is prospective and was approved by the Bioethics Committee of Beijing Friendship Hospital, Capital Medical University (ethics approval No. 2020-P2-021-02). all methods were carried out in accordance with relevant guidelines and regulations. This study was carried out in compliance with the STARD 2015.

All subjects signed informed consent forms prior to undergoing MRI.

## Materials and methods

### Patients

**Fig. 1:**
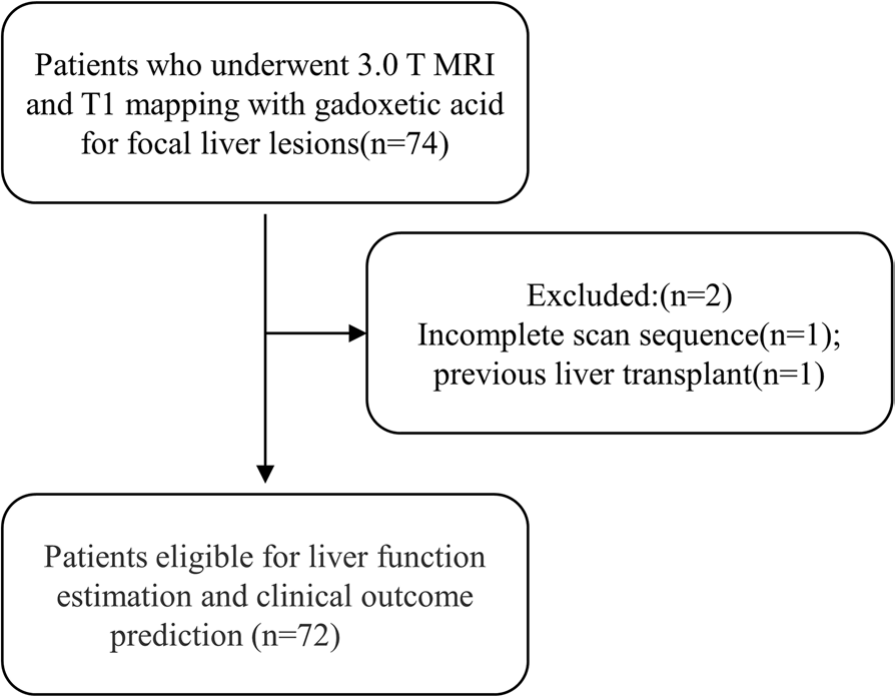
Flow chart of the study population

From August 2020 to March 2022, 74 patients underwent Gd-EOB-DTPA-enhanced MRI on a 3.0 T scanner to address concerns about focal liver lesions. Of these patients, 2 were excluded for the following reasons: 1 patient had an incomplete scan sequence, and 1 patient had undergone a liver transplant. Finally, 72 patients were included in this study (Fig. 1). Of these patients, there were 46 males and 26 females, with an average age of 52.81 years. Clinical data included serum total bilirubin (TBIL) level, albumin (ALB) level, prothrombin time (PT), creatinine level, and presence of ascites and hepatic encephalopathy. CP grade and ALBI grade of liver function were assessed according to the clinical data of each patient. ALBI grade was calculated as ALBI = -0.085 × albumin (g/L) + 0.66 × Log10 total bilirubin (μmol/L), and liver function grading was performed according to an established score [12].

According to the CP score, the patients were divided into a normal liver function group (NLF, *n* = 22), grade A group (LCA, *n* = 35), and grade B + C group (LCB + C, *n* = 15). According to the ALBI grade, the patients were divided into a normal liver function group (NLF, *n* = 22), grade 1 group (ALBI1, *n* = 18), and grade 2 + 3 group (ALBI2 + 3, *n* = 32).

### MRI

All patients underwent MRI on a 3.0 T unit (750 W, GE Healthcare, Milwaukee, WI, USA) using a 16-channel abdominal coil. In addition to routine imaging sequences, T1 mapping sequences were performed in the precontrast phase and at 20 min after contrast injection. The contrast agent was Gd-EOB-DTPA (Xianai; Chia Tai TianQing Pharma, LianYunGang, China), with an injection dose of 0.1 ml/kg and flow rate of 1 ml/s [13]. The T1 mapping sequence was performed with the variable-flip-angle technique, and the parameters were as follows: repetition time (TR) = 4.0 ms, echo time (TE) = 1.5 ms, matrix = 320 × 224, field of view = 380 mm × 296 mm, section thickness = 4 mm, and flip angles = 5°, 10° and 15°.

### Image measurements

**Fig. 2:**
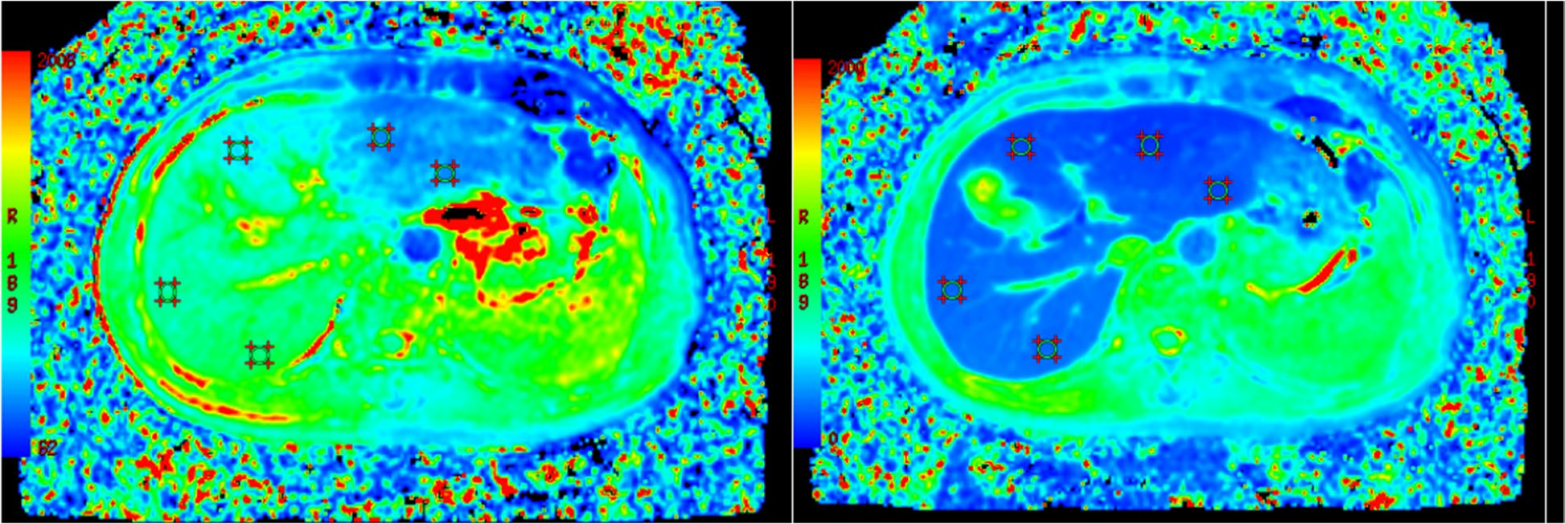
Images of the same patient Pre- and postcontrast T1 maps. The liver function assessment results placed the patient in the LCA group and the ALBI1 group. The left figure shows a plain scan, and the right image shows the scan acquired 20 min after Gd-EOB-DTPA injection. Five ROIs of equal size were drawn at the level of the porta hepatis to ensure that the ROI was at the same level and in the same position before and after enhancement as much as possible. The mean value was calculated as the T1 value of the whole liver

The T1 value was measured by two double-blinded radiologists with 10 years and 5 years of experience, respectively, with enhanced T1 maps before and after postprocessing, and then used the average as the final data. Five regions of interest (ROIs) of equal size were placed at the liver door level, and the ROIs were kept in the same position before and after enhancement while avoiding lesions, vasculature, bile ducts and artifacts. The average T1 values of the whole liver, T1 values before enhancement (T1pre), T1 values after enhancement (T1post) and rate of decrease in liver T1 relaxation time (ΔT1) were obtained. ΔT1 = (T1pre-T1post)/T1pre × 100% [14] (Fig. 2).

### Statistical methods

The chi-square test and one-way ANOVA were used to compare clinical data between groups. The intraclass correlation coefficient (ICC) was used to evaluate the consistency of the measurement results among physicians. Spearman rank correlation analysis was used to assess the correlation of each index with the C-P grade and ALBI grade. Pearson analysis was used to evaluate the correlations of T1 mapping parameters with the levels of ALB and TBIL and the PT in patients with abnormal liver function. A nonparametric test was used to compare T1pre, T1post and ΔT1 between different groups in terms of the C-P and ALBI grades. Receiver operating characteristic (ROC) curves were used to evaluate the liver function classification efficiency of each imaging index. *P* < 0.05 was considered statistically significant.

## Results

### Clinical data and laboratory examinations

The laboratory parameters and clinical data of the 72 included subjects are shown in Table 1. The creatinine values of all the patients were within the normal range, and no hepatic encephalopathy was assessed clinically. There were statistically significant differences in age and sex among all the groups (*p* < 0.05), and there were significant differences in the levels of TBIL and ALB, PT and presence of ascites among all the analyzed groups (*p* < 0.001).

**Table 1:** The laboratory parameters and clinical data of the patients

**Characteristic**	**Total**	**Normal**	**LCA**	**LCB + C**	**ALBI1**	**ALBI2 + 3**	***P* value (C-P/ALBI)**
Sample size	72	22	35	15	18	32	-
Age (years)^a^	52.81 ± 13.69	44.72 ± 13.41	55.57 ± 12.89	58.20 ± 11.12	50.05 ± 11.62	60.23 ± 11.27	< 0.05/ < 0.05
Sex (male/female)	46/26	8/14	29/6	9/6	16/2	22/10	< 0.05/ < 0.05
TBIL (μmol/L)^a^	24.09 ± 19.46	12.10 ± 8.33	19.77 ± 8.34	47.78 ± 26.72	18.62 ± 7.88	34.03 ± 23.58	< 0.001/ < 0.001
ALB (g/L)^a^	39.19 ± 5.67	41.525 ± 4.54	41.00 ± 4.63	32.65 ± 3.79	43.94 ± 3.35	35.15 ± 4.28	< 0.001/ < 0.001
PT (s)^a^	13.01 ± 2.81	10.48 ± 4.27	12.38 ± 1.13	15.96 ± 2.46	12.10 ± 1.08	14.22 ± 2.48	< 0.001/ < 0.001
Ascites (no/yes)	51/21	22/0	27/8	2/13	15/3	14/18	< 0.001/ < 0.001

*PT* Prothrombin time, *TBIL* Total bilirubin, *ALB* Albumin

^a^Data are presented as the mean (± standard deviation)

### Consistency of measurement results

The measurement results were consistent between the two physicians, with ICC values of 0.923 (95% CI: 0.881–0.951) and 0.976 (95% CI: 0.963–0.985) for T1pre and T1post, respectively.

### Correlation between T1 mapping parameters and liver function grade

**Fig. 3:**
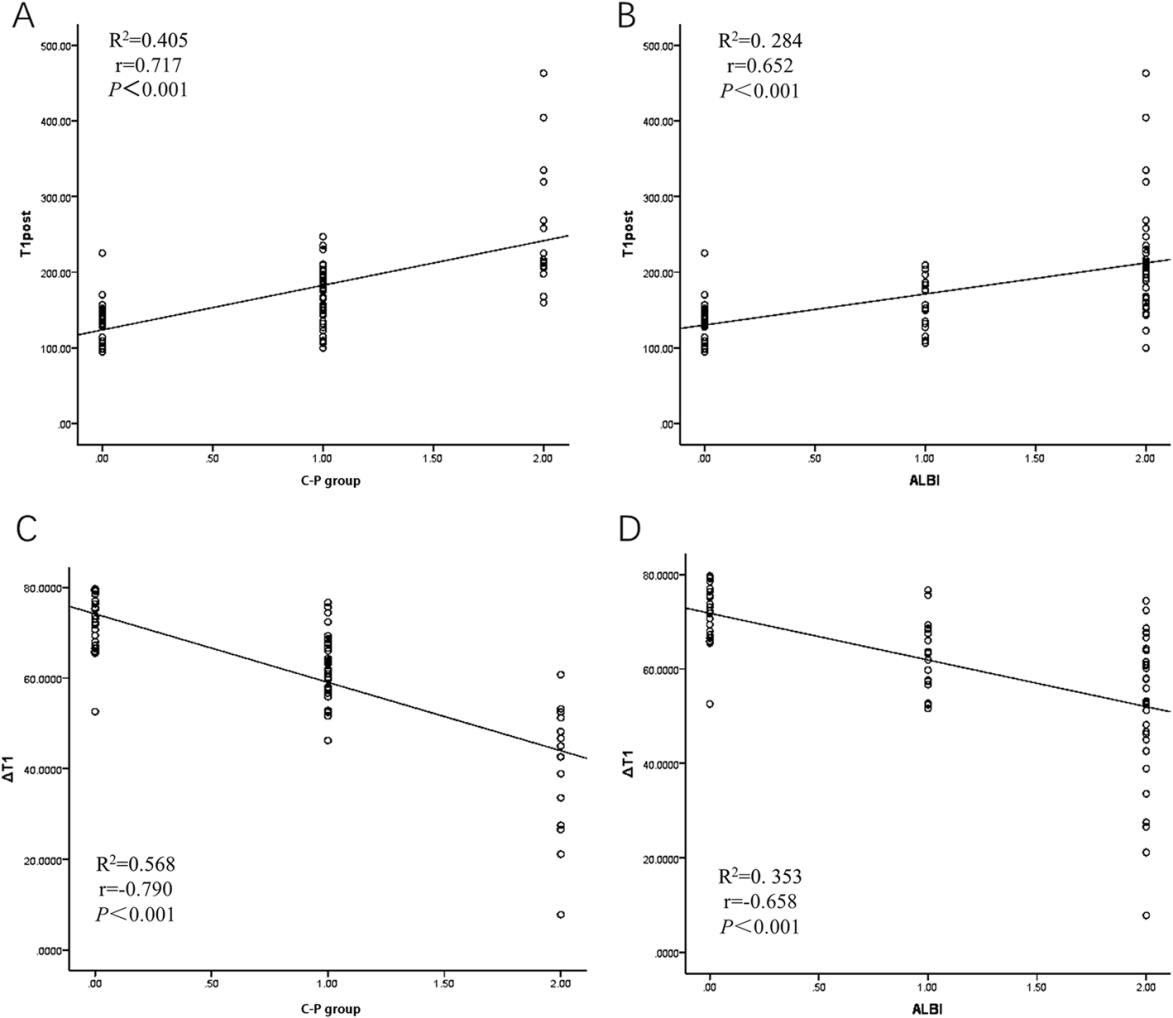
**a** and **b** show that T1post is positively correlated with the C-P grade and the ALBI grade. **c** and **d** show that ΔT1 was negatively correlated with both the C-P grade and the ALBI grade

**Fig. 4:**
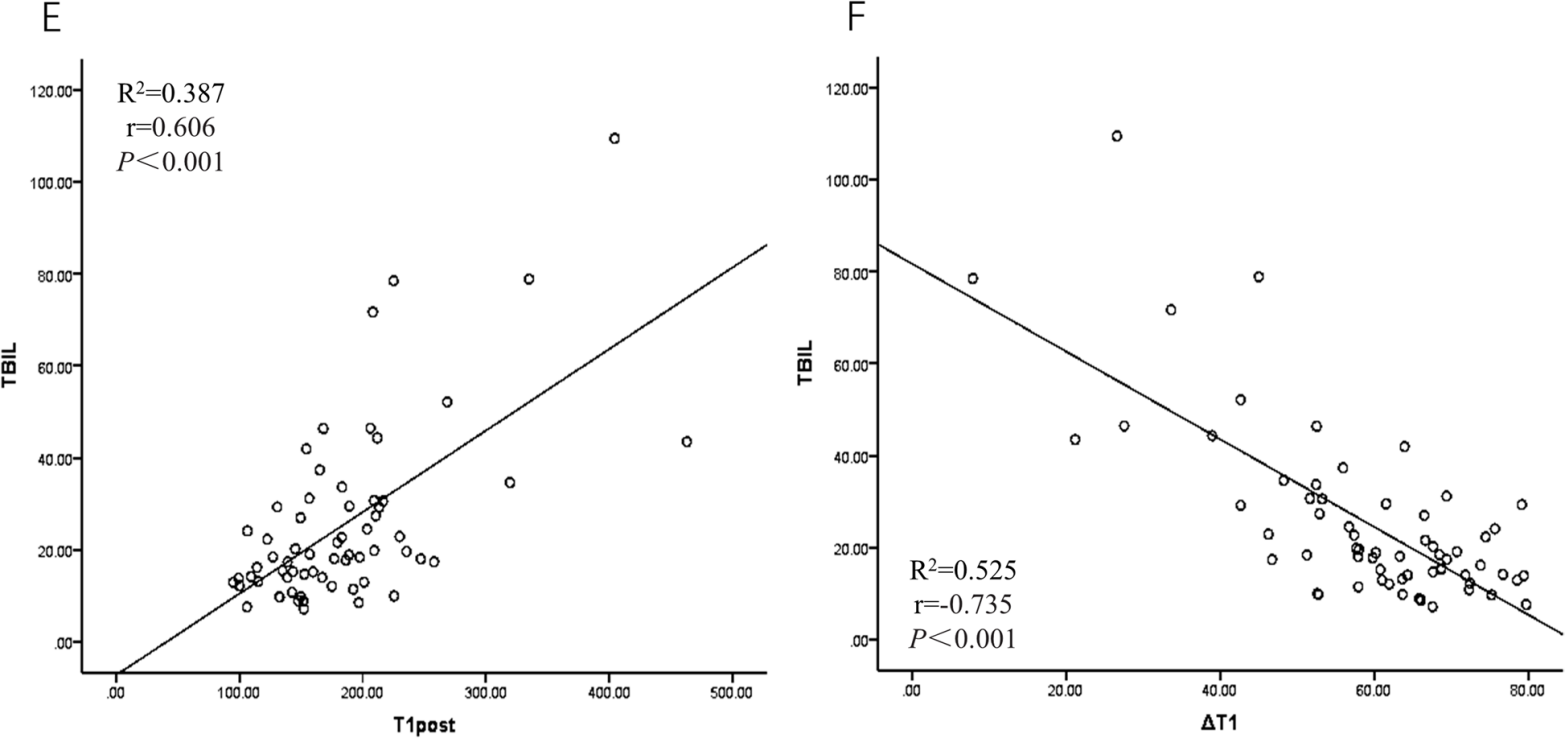
**e** shows that TBIL was positively correlated with T1post. **f** shows a negative correlation between TBIL and ΔT1

1. T1post was positively correlated with the C-P grade and the ALBI grade (*r* = 0.717 and *r* = 0.652) and increased gradually with the severity of liver function impairment. ΔT1 was negatively correlated with both the C-P grade and the ALBI grade (*r* = -0.790 and -0.658) and decreased gradually with the severity of liver function impairment (Fig. 3). T1pre was not significantly correlated with the C-P grade or the ALBI grade (*p* > 0.05).
2. In patients with abnormal liver function, Pearson correlation analysis of T1 mapping parameters with ALB, TBIL and PT showed that TBIL had the strongest correlation with the T1 mapping parameter. The level of TBIL was moderately positively correlated with T1post and had a moderate negative correlation with ΔT1, with correlation coefficients of *r* = 0.606 and *r* = -0.735, respectively (Fig. 4). The level of ALB was a significant factor influencing the ALBI grade (*p* < 0.01), with a correlation coefficient of *r* = -0.788. The correlation between the level of TBIL and the ALBI grade was weak, with a correlation coefficient of *r* = 0.329.

### Comparison of T1 mapping parameters among different liver function grades

**Fig. 5:**
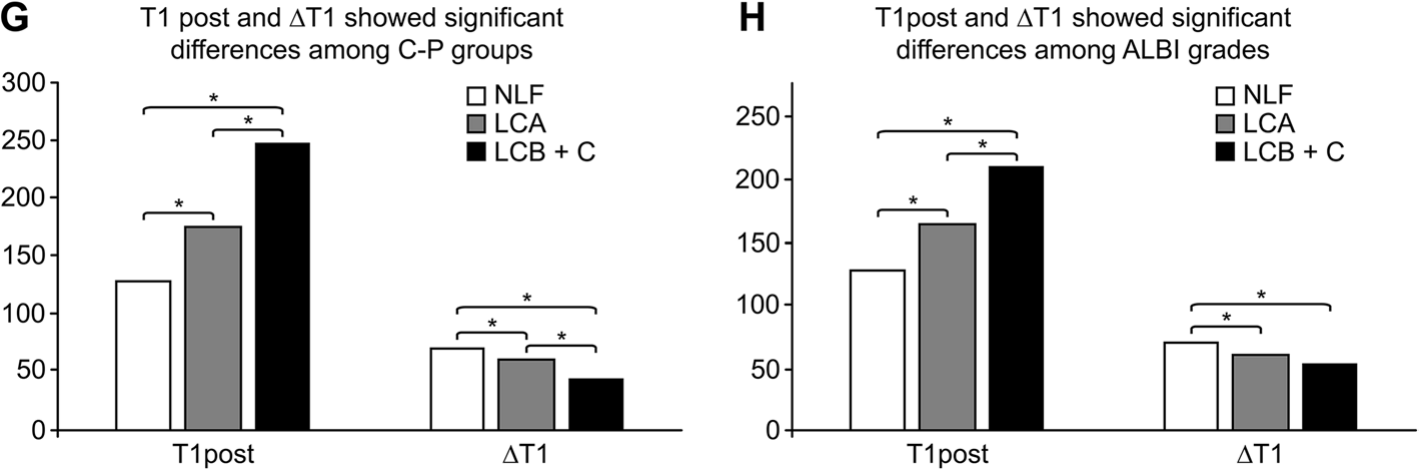
Double arrows indicate a significant difference between the two groups (*p* < 0.05). **g** shows that T1post and ΔT1 were significantly different in pairwise comparisons between the C-P groups. **h** shows that T1post was significantly different in pairwise comparisons among the ALBI grade groups, while ΔT1 was not significantly different between the AlBI1 and AlBI2 + 3 groups

There were significant differences in T1post and ΔT1 among the different liver function grades (*p* < 0.05). Pairwise comparisons between groups showed that T1post and ΔT1 had significant differences among different C-P grades (*p* < 0.05). Regarding the ALBI grade, T1post showed statistically significant differences among all groups (*p* < 0.05), while ΔT1 only showed statistically significant differences between the NLF and ALBI1 groups, without significant differences between the ALBI1 and ALBI2 + 3 groups (Table 2 and Fig. 5).

**Table 2:** Comparison of T1post and ΔT1 among the C-P grades and ALBI grades ($\overline{x }\pm s$)

**Group**	**Sample size**	**T1post (ms)**	**ΔT1 (%)**
C-P group			
NLF	22	134.202 ± 29.423	71.375 ± 6.443
LCA	35	169.516 ± 37.770	62.585 ± 7.199
LCB + C	15	257.009 ± 87.330	39.925 ± 14.158
*F*		32.517	41.607
*P* value		< 0.001	< 0.001
ALBI group			
NLF	22	134.202 ± 29.423	71.375 ± 6.443
ALBI1	18	161.560 ± 34.667	62.960 ± 7.417
ALBI2 + 3	32	215.003 ± 76.689	51.747 ± 15.638
*F*		30.471	31.798
*P* value		< 0.001	< 0.001

### ROC analysis

**Fig. 6:**
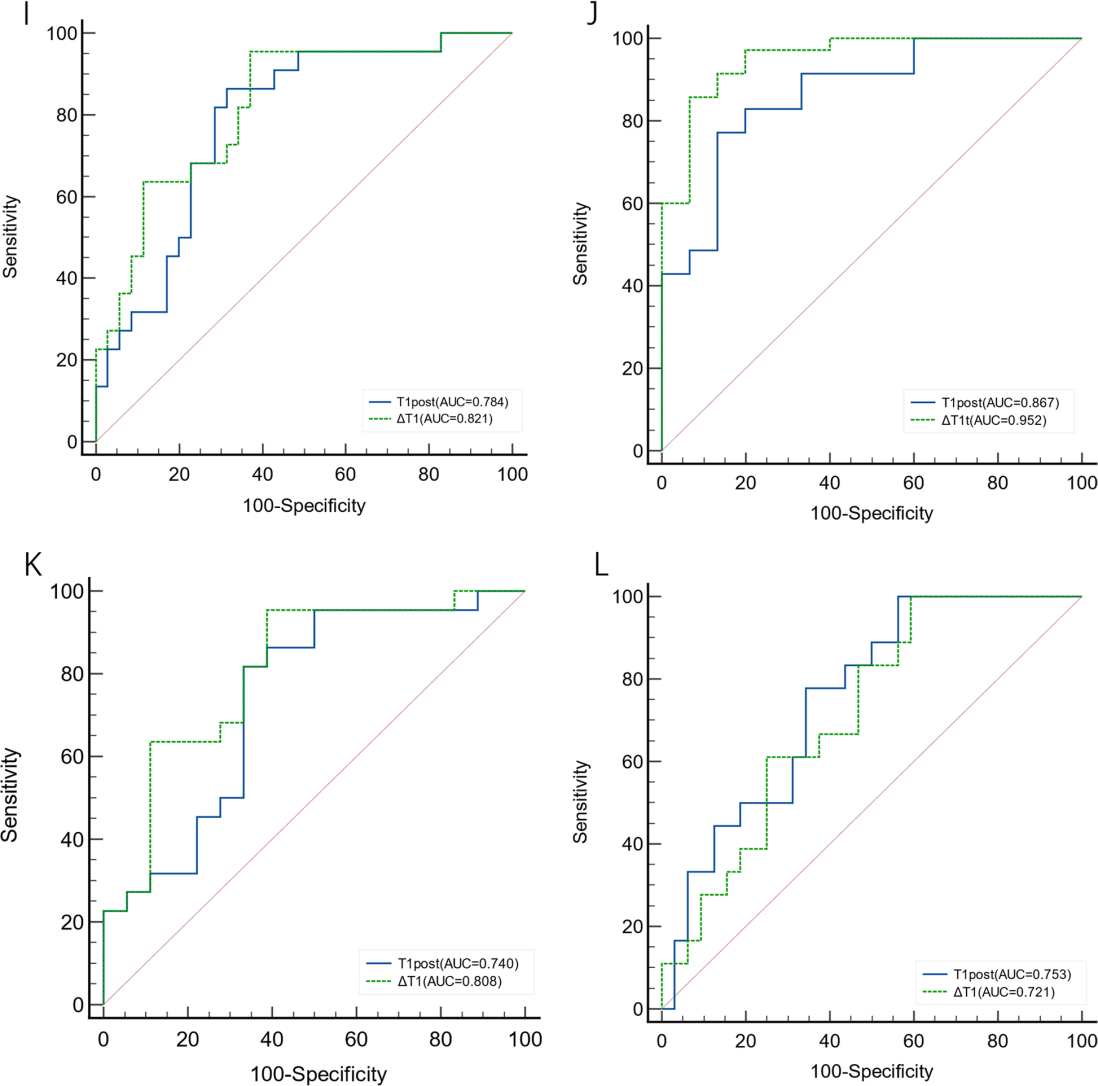
ROC curve of T1post and ΔT1 in differentiating between groups according to liver function. **i** shows ROC curve of between the NLF and LCA groups. **j** shows ROC curve of between the LCA and LCB + LCC groups. **k** shows ROC curve of between the NLF and ALBI 1 groups. **l** shows ROC curve of between the ALBI 1 and ALBI2 + 3 groups

T1post and ΔT1 had better diagnostic efficacy in differentiating different groups of liver function based on the C-P grade, with ΔT1 being slightly better than T1post. The AUCs for differentiating between the NLF and LCA groups and between the LCA and LCB + C groups were 0.821 and 0.952, respectively. For the ALBI grade, T1post showed good ability to distinguish between the NLF and ALBI1 groups and between the ALBI1 and ALBI2 + 3 groups, with a sensitivity of 100% and AUCs of 0.740 and 0.753, respectively. ΔT1 was only good at differentiating between the NLF and ALBI1 groups, with a sensitivity of 95.5% and AUC of 0.808. This parameter had an insufficient ability to discriminate between the ALBI1 and ALBI2 + 3 groups (Table 3 and Fig. 6).

**Table 3:** The diagnostic efficacy of T1post and ΔT1 in differentiating between groups according to liver function

	**C-P**				**ALBI**			
	**NLF from LCA**		**LCA from LCB + C**		**NLF from ALBI1**		**ALBI1 from ALBI2 + 3**	
	**T1post**	**ΔT1**	**T1post**	**ΔT1**	**T1post**	**ΔT1**	**T1post**	**ΔT1**
Sensitivity	86.4	95.5	77.1	85.7	81.8	95.5	100.0	100.0
Specificity	68.6	62.9	86.7	93.3	66.7	61.1	43.7	40.6
Cutoff value	151.821	64.310	196.833	53.242	149.525	63.690	209.393	51.246
AUC	0.784	0.821	0.867	0.952	0.740	0.808	0.753	0.721
*P*	< 0.001	< 0.001	< 0.001	< 0.001	0.004	< 0.001	< 0.001	0.014

## Discussion

Gd-EOB-DTPA is a hepatocyte-specific contrast agent. After being absorbed by normal liver cells, approximately 50% of the agent is excreted into the biliary system via multidrug resistant protein 2 (MRP2) on the hepatocyte membrane, and the rest is excreted through the kidney. Its uptake in liver cells has been believed to be mediated by passive diffusion of organic anion transporter polypeptide 1 (OATP1) expressed on the liver membrane [15]. The contrast agent has been associated with a shortened liver T1 relaxation time. Since the absorption of disodium gadolinium is dependent on hepatocyte integrity, this absorption can be quantified to assess liver function [16]. The correlation analysis results of this study showed that bilirubin was moderately correlated with T1 mapping parameters. Ute Lina Fahlenkamp et al. [17] showed a moderate positive association between bilirubin and T1post, which is consistent with our findings. The uptake and excretion of bilirubin in liver cells involves OATP1 and MRP2, respectively [18], and the transporter of bilirubin in the liver membrane is the same as that of disodium gadolinium. The expression of OATPs on the surface of the liver cell membrane decreases due to liver fibrosis or cirrhosis [19], and bilirubin and gadolinium disodium compete for this transporter, resulting in reduced uptake of gadolinium disodium by liver cells and a decrease in the T1post value in the liver [20].

In clinical practice, patients with a C-P grade higher than grade B are considered to have a higher risk for surgery, so in this study, grade B and C liver function were combined into one group. Our results showed that T1pre was not significantly correlated with the C-P grade of liver function, This is consistent with the findings of Li Jiamin et al. [21], due to the influence of many factors common in hepatitis and cirrhosis, including varying degrees of liver fibrosis, metal deposition, and fatty infiltration. While T1post and ΔT1 were highly correlated with the C-P grade, which was consistent with the results of Pan et al. [22]. T1post and ΔT1 were significantly associated with the C-P classification of liver function. T1post increased gradually with the severity of liver function impairment, while ΔT1 decreased gradually with higher severity. Pairwise comparisons between the groups showed statistically significant differences, which was consistent with the report from Yu et al. [14]. Verena Carola Obmann et al. [23] also suggested that ΔT1 distinguished patients without cirrhosis from those with cirrhosis, and that ΔT1 was a good predictor of cirrhosis C-P grading. In this study, the diagnostic efficacy of ΔT1 was slightly higher than that of T1post in the pairwise comparisons between C-P groups. The sensitivity of ΔT1 in differentiating between the NLF and LCA groups was as high as 95.5%, cutoff of with 64.310%, and this parameter could accurately identify abnormal liver function in the tested population. The specificity of this parameter for distinguishing between the LCA and LCB + C groups reached 93.3%, cutoff of with 53.242%, showing that ΔT1 can effectively reduce the misdiagnosis rate of patients with decompensated liver function and provide more accurate information for the preoperative evaluation of patients with abnormal liver function.

In recent years, the ALBI grade has been proposed as a method to evaluate liver function. This association was originally established by studying the survival rate of patients with a high risk of hepatocellular carcinoma (HCC), and this parameter can play an important role in the detailed assessment of relative changes in liver function during treatment [12]. The ALBI grade only involves two factors, total bilirubin and albumin, and is not affected by subjective judgment. Studies have shown that HCC patients classified with an ALBI grade of 1 undergoing microwave ablation (MWA) have better overall and disease-free survival than those with higher ALBI grades [24]. King et al. [25] reported that sorafenib had limited efficacy in HCC patients with an ALBI grade ≥ 2, so this study divided ALBI grades 2 + 3 into one group. Davide Ippolito et al. [26] suggested that there was no statistically significant difference in the ratio of liver-muscle signal intensity in the hepatobiliary stage among the ALBI grading groups. Few studies have examined whether there is a relationship between T1 mapping parameters and the ALBI grade. Our results show that T1post and ΔT1 significantly differ between ALBI grading groups. T1post and ΔT1 could be used to distinguish between the NLF and ALBI1 groups with a sensitivity greater than 80%; moreover, the AUC value of ΔT1 was higher than that of T1post. The sensitivity of T1post was high in distinguishing between the AlBI1 and AlBI2 + 3 groups, but the difference in ΔT1 between these groups was not statistically significant, which may be due to the partial nonoverlap between the ALBI and C-P classifications. The ALBI2 group included a larger range of C-P grades, with some patients from the LCA group and one patient classified as LCC being assigned to this group. The correlation analysis results of patients with abnormal liver function showed that albumin was a significant factor influencing the ALBI grade, while total bilirubin had a relatively weak influence, and the correlation between T1mapping parameters and albumin was lower than that of total bilirubin. In addition, this grading method did not include clotting-related indicators. Cheng et al. [27] believed that the PT was an independent risk factor for predicting the degree of liver fibrosis.

The limitations of this study are as follows: first, the sample size of this study was small, especially the number of patients with decompensated cirrhosis. In addition, CP grades 2 and 3 as well as ALBI grades 2 and 3 were combined into the LCB + C and ALBI2 + 3 groups, and the ability of T1 mapping to differentiate between moderately and severely impaired liver function was not analyzed. Second, the study included patients with cirrhosis of various etiologies without further analysis. Third, this is a single-center study with only internal validation, so its conclusions need external validation and multicenter prospective cohort validation to be used in clinical practice. Finally, the study focused on evaluations of the whole liver; future studies should focus on combining these evaluations with assessments of liver segments.

In conclusion, the T1post and ΔT1 values of Gd-EOB-DTPA-enhanced MRI with T1 mapping can be used as noninvasive imaging indicators for evaluating liver function. These parameters can meet the requirements for imaging diagnosis and be used to effectively monitor changes in liver function grade during preoperative evaluations and postoperative follow-up.

## Data Availability

The datasets used during the current study are available from the corresponding author on reasonable request.
